# Association between medical insurance type and survival in patients undergoing peritoneal dialysis

**DOI:** 10.1186/s12882-015-0023-7

**Published:** 2015-03-21

**Authors:** Zengsi Wang, Yanmin Zhang, Fei Xiong, Hongbo Li, Yanqiong Ding, Yihua Gao, Li Zhao, Sheng Wan

**Affiliations:** Department of nephrology, Wuhan No.1 hospital, 430022 Wuhan, Hubei Province China

**Keywords:** Peritoneal dialysis, Medical insurance, Survival, Rural, Urban

## Abstract

**Background:**

Socioeconomic characteristics may affect the outcomes of patients treated with peritoneal dialysis (PD). There are two major medical insurances in China: the New Cooperative Medical Scheme (NCMS), mainly for rural residents, and the Urban Employees’ Medical Insurance (UEMI). The aim of the present study was to assess the effect of medical insurance type on survival of patient undergoing PD.

**Method:**

This was a prospective study in adult patients who underwent PD at the Wuhan No.1 Hospital between January 2008 and December 2013. Patients had received continuous ambulatory PD for >3 months. Patients were divided according to their medical insurance. Demographic and socioeconomic data, biochemical parameters and primary clinical outcomes including all-cause mortality, switch to hemodialysis and kidney transplantation were analyzed.

**Result:**

There were 415 patients with UEMI and 149 with NCMS. Compared with UEMI, patients with NCMS were younger, and had shorter dialysis duration, smaller proportion of diabetic nephropathy, more severe anemia, and more frequent hyperphosphatemia and hyperuricemia. Total Kt/V, creatinine clearance and residual renal function were not different. There was no difference in technique survival (P > 0.05) between the two groups, but rural patients showed lower overall survival (P < 0.05). Multivariate analysis showed that NCMS was independently associated with lower survival (RR = 1.49; 95% CI = 1.04-2.15).

**Conclusions:**

Medical insurance model is independently associated with PD patient survival.

## Background

The association between patient characteristics and clinical outcomes in patients treated with peritoneal dialysis (PD) are complex and multifactorial [1-3]. Previous studies have shown that characteristics such as lower income, lower education level and being female were associated with higher mortality in PD patients [4-6]. In addition, dialysis patients living in rural areas have higher mortality compared with urban residents [7]. On the other hand, the patient volume of the PD center, education level and the distance from the residence to the hospital are associated with peritonitis, the most important complication of PD [8,9]. In addition, black race, diabetes and advanced age are predictors of peritonitis occurrence [9,10]. Racial and insurance disparity may affect the choice of treatment for patients with end-stage renal disease (ESRD) [11]. However, another study has shown that the economic status was not independently associated with outcomes in a large multicenter Brazilian cohort [12].

Approximately of 50% of Chinese people lives in rural areas, and these areas are often underdeveloped and far from hospitals. A previous study has shown that living far from the PD unit was associated with shorter time to first peritonitis episode, but was independently predictive of higher rate of cure with antibiotics alone and trends to lower rates of catheter removal and permanent switch to hemodialysis (HD) [13]. Another study has shown that low personal income influenced all-cause and cardiovascular survival, and initial peritonitis in PD patients, whiles education level predicted all-cause survival only for patients living in underdeveloped regions [14].

In China, the two major medical insurance systems are the New Cooperative Medical Scheme (NCMS) for rural residents, and the Urban Employees’ Medical Insurance (UEMI) for urban patients. However, there are many disparities in the funding source, financing level and proportion of reimbursement between the two insurance systems [15]. Even if people participating in the NCMS are less likely to become impoverished, NCMS reimbursement policies are far from optimal, which may interfere with the choice of treatment and compliance [16]. However, there is no study about the association between disparities in medical insurance and PD outcomes in China.

The present study examined the association between insurance type and outcomes after initiation of PD in a large volume PD center. Results of the present study might provide a rationale for individualization and tailoring of the therapeutic approach, particularly for rural patients.

## Methods

### Participants

This was a prospective observational cohort study in all patients from the PD center of the Wuhan No.1 Hospital (Wuhan, China) receiving PD between January 2008 and December 2011. Patients were followed up until death or December 31^st^, 2013 (the end of the study), after which survival data were censored. Inclusion criteria were: 1) patients with ESRD; 2) aged 18-80 years; 3) received continuous ambulatory PD for more than 3 months; and 4) provided complete information about their socioeconomic status. Exclusion criteria were: 1) malignant disease; 2) refused to provide written consent; 3) unavailable or incomplete data about clinical and laboratory examination; or 4) the patient had no insurance. There is no automated PD in our center.

This study was approved by the ethical committee of the Wuhan No.1 Hospital and all participants provided a written informed consent.

### Data collection

Data were obtained from patient records and medical staff reports. The demographic and socioeconomic data included family income, educational level (illiteracy, defined as less than elementary school, or literacy, defined as elementary school graduate or higher), etiology of chronic kidney disease (CKD), body mass index (BMI) and blood pressure (BP). Biochemical parameters including hemoglobin, serum albumin, serum creatinine, serum uric acid, albumin-corrected calcium, serum phosphorus and parathyroid hormone were measured at the central laboratory of the Wuhan No.1 Hospital. All biochemical parameters were measured at least yearly in all patients after PD treatments, and the average for each parameter was used in our analyses. Residual kidney function was assessed using glomerular filtration rate (GFR). Total Kt/V was calculated using the PD Adequest software 2.0 (Baxter, Deerfield, IL, USA). GFR, in ml/min/1.73 m^2^, was estimated from the mean values of creatinine clearance (CrCL) and urea clearance, and adjusted for body surface area [17].

### Clinical outcomes

The primary clinical outcomes were all-cause mortality, switch to HD and kidney transplantation. Causes of death were grouped as: 1) cardiovascular death (myocardial infarction, cardiac arrest, heart failure, cerebrovascular accident, and other cardiac causes); 2) peritonitis-related mortality; 3) treatment withdrawal for financial reasons (gave up treatment if the family could not afford high medical expenses); and 4) other or unknown causes. Causes of switch to HD were grouped as: 1) peritonitis; 2) inadequate dialysis (including ultrafiltration failure and other medical causes); and 3) others (catheter-related complications or medical problems such as pleural effusions). For the purposes of this study, all outcomes data (survival, transplantation, withdrawal, technique failure, etc.) for all patients were censored on December 31^st^, 2013.

### Statistical analyses

Continuous variables are expressed as mean ± SD for normally distributed data, or as median and frequency (%) for non-normally distributed data. Categorical data are presented as proportions. Differences in demographics, clinical characteristics and laboratory parameters between the two groups were analyzed using the chi-square test for categorical data, and the unpaired t-test for normally distributed continuous data. Survival curves and survival probabilities were generated using the Kaplan-Meier method. Risk factors associated with mortality and technique failure were determined by multivariate Cox proportional hazards model. The covariates included in the Cox regression models were age (as a continuous variable), sex, education, insurance, BMI and laboratory data (listed in Table 1). Statistical analysis was performed using SPSS 17.0 (IBM, Armonk, NY, USA). Statistical significance was defined as P < 0.05.

**Table 1:** **Biochemical characteristics of the study patients (data are averages over the duration of the study)**

**Characteristics**	**Urban medical insurance (n = 415)**	**Rural medical insurance (n = 149)**	**P-value**
HGB (g/L)	95.19 ± 21.13	89.83 ± 23.98	0.011
FERR (μg/L)	201.97 ± 209.17	232.33 ± 236.90	0.178
Ca (mmol/L)	2.15 ± 0.25	2.06 ± 0.25	<0.001
P (mmol/L)	1.69 ± 0.52	1.81 ± 0.53	0.019
PTH (pg/ml)	459.93 ± 475.38	468.95 ± 433.90	0.845
ALB (g/L)	36.33 ± 4.61	36.39 ± 5.06	0.900
BUN (mmol/L)	18.37 ± 13.16	19.21 ± 7.98	0.464
Cr (μmol/L)	878.31 ± 288.66	923.03 ± 314.03	0.114
UA (mmol/L)	413.09 ± 84.63	431.68 ± 87.35	0.023
nPCR (g/kg/d)	0.95 ± 0.23	0.95 ± 0.21	0.778
D/P at 4 hours	0.66 ± 0.14	0.68 ± 0.15	0.156
Kt/V	1.75 ± 0.55	1.68 ± 0.59	0.164
CrCL (L/W)*	61.28 ± 35.65	59.20 ± 22.91	0.506
GFR^#^	1.88 ± 2.64	2.14 ± 2.46	0.297
PD volume (ml)	7228.16 ± 1546.17	6572.41 ± 1484.85	<0.001

Data are presented as mean ± SD or median (range).

HGB: hemoglobin; FERR: Ca: calcium; P: phosphorus; PTH: parathyroid hormone; ALB: albumin; BUN: blood urea nitrogen; Cr: creatinine; UA: uric acid; nPCR: normalized protein catabolic rate; D/P: dialysate/plasma creatinine; Kt/V: dialysis efficiency; CrCL: creatinine clearance rate; GFR: glomerular filtration rate; PD: peritoneal dialysis.

*Adjusted for BSA.

^#^GFR, in ml/min/1.73 m^2^, was estimated from the mean values of creatinine clearance and urea clearance, and adjusted for body surface area [17].

## Results

### Demographic and clinical characteristics

A total of 564 patients were included: 415 (77.0%) with UEMI and 149 (23.0%) with NCMS. Baseline demographic and clinical characteristics are presented in Table 2. Compared with patients with UEMI, patients with NCMS were younger (51.9 ± 14.7 vs. 57.3 ± 13.2 years, P < 0.001), had a shorter dialysis duration (time from the first PD treatment to study recruitment; 22.8 ± 17.6 vs. 31.4 ± 25.8 months, P = 0.01), had a smaller proportion of diabetic nephropathy (DN) (6.0% vs. 16.1%, P = 0.02) and had a higher proportion of illiteracy (71.1% vs*.* 44.1%, P < 0.001). There was no difference in sex, BMI and systolic BP between the two groups.

**Table 2:** **Demographic and baseline characteristics of the patients**

**Characteristics**	**Urban medical insurance (n = 415)**	**Rural medical insurance (n = 149)**	**P-value**
Age (yr)	57.34 ± 13.21	51.85 ± 14.65	<0.001
Male, n (%)	209 (50.4%)	89 (49.6%)	0.677
Education level, n (%) Illiteracy Literacy	183 (44.1%) 232 (55.9%)	106 (71.1%) 43 (28.9%)	<0.001
DN, n (%)	67 (16.1%)	9 (6.0%)	0.02
Dialysis duration* (months)	31.40 ± 25.80	22.84 ± 17.59	0.01
Body weight (kg)	60.28 ± 11.01	60.35 ± 10.18	0.947
BMI (kg/m^2^)	22.46 ± 3.38	22.26 ± 3.04	0.531
SBP (mmHg)	137.56 ± 16.92	139.24 ± 18.10	0.411
DBP (mmHg)	82.46 ± 10.54	86.47 ± 12.23	0.002

DN: diabetic nephropathy; BMI: body mass index; SBP: systolic blood pressure; DBP: diastolic blood pressure.

*Time from the first PD treatment to study recruitment.

### Biochemical parameters

During follow-up, patients with NCMS showed lower iron levels (89.83 ± 23.98 vs. 95.19 ± 21.13 g/L, P = 0.01), higher phosphorus levels (1.81 ± 0.53 vs. 1.69 ± 0.52 mmol/L, P = 0.02), and lower calcium levels (2.06 ± 0.25 vs. 2.15 ± 0.25 mmol/L, P < 0.001) compared with patients with UEMI. There was no difference in albumin, creatinine, urea nitrogen and normalized protein catabolic rate (nPCR) between the two groups (Table 1).

### PD prescription and adequacy data

During follow-up, despite rural patients having a smaller PD volume (6572 ± 1484 vs*.* 7228 ± 1546 ml, P < 0.001), comparison of adequacy data showed that total Kt/V and CrCL were not different, and that residual GFR was not different between the two groups (Table 2).

### Clinical outcome

Among all patients, 173 (41.7%) patients with UEMI and 58 (38.9%) patients with NCMS ceased treatment before the end of the study. There was no difference in unadjusted death rates between groups on a univariate analysis. Death was the main cause of treatment cessation, and there was no significant difference between the two groups (21.9% vs*.* 26.2%, P = 0.29). However, switch to HD was less frequent in patients with NCMS (15.9% vs. 8.1%, P = 0.02). Patients with NCMS had a shorter time on therapy compared with patients with UEMI (27.4 ± 19.9 vs*.* 36.6 ± 25.7 months, P = 0.01; unadjusted data) (Table 3).

**Table 3:** **Causes for ceasing peritoneal dialysis**

**Characteristics**	**Urban medical insurance (n = 415)**	**Rural medical insurance (n = 149)**	**P-value**
Death, n (%)	91 (21.9%)	39 (26.2%)	0.291
Switch to HD, n (%)	66 (15.9%)	12 (8.1%)	0.017
Transplant, n (%)	15 (3.6%)	5 (3.4%)	0.884
Others, n (%)	1 (0.2%)	2 (1.3%)	0.113
TOT (month)	36.55 ± 25.74	27.36 ± 19.88	0.014

HD: hemodialysis; TOT: time on treatment.

### Predictors of technique failure

During follow-up, 78 patients switched to HD. Peritonitis was the main cause of technique failure in both groups, followed by inadequate dialysis. The distribution of different causes of technique failure was similar between the two groups (Table 4; unadjusted data).

**Table 4:** **Causes of switch to HD**

**Characteristics**	**Urban medical insurance (n = 66)**	**Rural medical insurance (n = 12)**	**P-value**
Peritonitis, n (%)	48 (72.7%)	7 (58.3%)	0.314
Inadequate dialysis, n (%)	5 (7.6%)	1 (8.3%)	0.928
Others, n (%)	13 (19.7%)	4 (33.3%)	0.293

**Figure 1:**
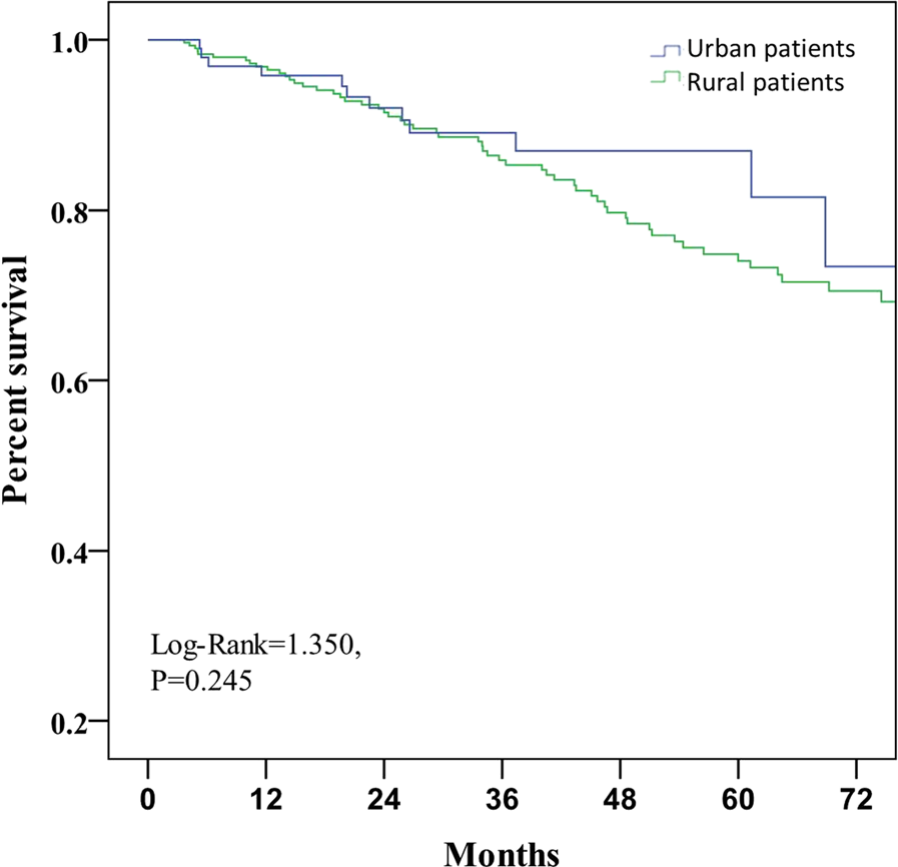
**Patient PD technique survival for urban and rural patients.**

The technique survival rates at 1, 2, 3 and 5 years were 96.5%, 91.4%, 85.9% and 73.3%, respectively, for patients with NCMS, and 95.8%, 92.0%, 87.0% and 81.5%, respectively, for patients with UEMI (P = 0.245) (Figure 1). After adjustment for demographic and clinical characteristics, Cox model analysis showed that diabetic nephropathy (relative risk (RR) = 1.85, 95% confidence interval (95% CI): 1.10-3.13) and lower albumin (RR = 0.90, 95% CI: 0.86-0.94) were independent predictors of technique failure in all patients.

### Patient survival and predictors of mortality

**Figure 2:**
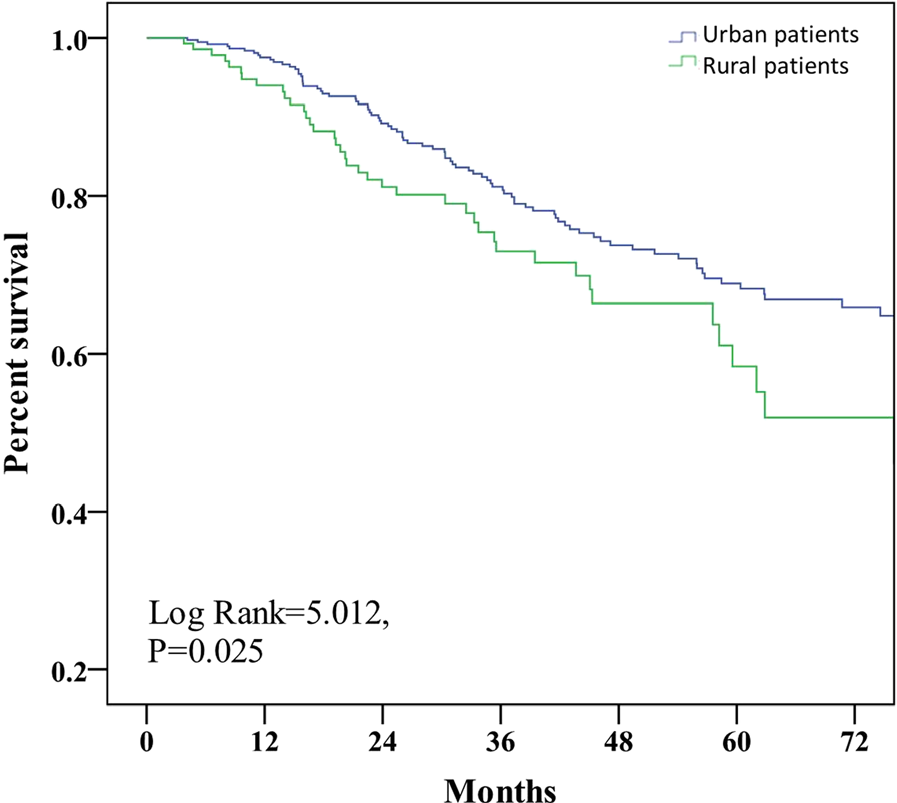
**Patient survival for urban and rural PD patients.**

In the present study, patient survival according to insurance type was the primary outcome. Cardiovascular diseases were the main cause of death in both groups (25.6% vs. 35.2%, P = 0.29). Treatment withdrawal for financial reasons was more frequent in patients with NCMS compared with patients with UEMI (15.4% vs. 1.1%, P = 0.001) (Table 5). The unadjusted patient survival rate at 1, 2, 3 and 5 years were 94.0%, 81.1%, 73.0% and 58.4%, respectively, in patients with NCMS, and was 97.5%, 89.2%, 81.2% and 68.9%, respectively, in patients with UEMI. Kaplan-Meier analysis showed a higher survival in patients with UEMI (*P* < 0.05) (Figure 2). The relative risk of death for rural patients was 1.49 (95% CI, 1.04-2.15). Multivariate Cox regression analysis showed that education level, insurance type, age, albumin, serum phosphorus, peritoneal equilibration test and total Kt/V were independent predictors of patient survival in the combined cohort (Table 6).

**Table 5:** **Causes of mortality**

**Causes of death**	**Urban medical insurance (n = 91)**	**Rural medical insurance (n = 39)**	**P-value**
CVD, n (%)	32 (35.2%)	10 (25.6%)	0.287
Peritonitis, n (%)	17 (18.7%)	3 (7.7%)	0.112
Financial reasons, n (%)	1 (1.1%)	6 (15.4%)	0.001
Other, n (%)	41 (45.1%)	20 (51.3%)	0.514

CVD: cardiovascular death.

**Table 6:** **Predictors of mortality on Cox multivariate analysis**

**Characteristics**	**P value**	**RR**	**95% CI**
Education level	<0.01	0.52	0.35, 0.76
Insurance model	0.03	1.49	1.04, 2.15
Albumin	0.01	0.95	0.91, 0.98
Age	<0.01	1.03	1.02, 1.04
Kt/V	0.01	0.64	0.46, 0.89
Peritoneal equilibrium test	<0.01	0.21	0.11, 0.40
Phosphorus	0.04	1.41	1.02, 1.95

Kt/V: dialysis efficiency; RR: relative risk; 95% CI: 95% confidence interval.

## Discussion

The present study was a prospective analysis of the relationship between clinical outcomes of PD and medical insurance model. Data showed that patients with NCMS had more severe anemia and hyperphosphatemia, and that survival was significantly lower in patients with NCMS compared with patients with UEMI. However, the technique failure rate was similar between the two groups.

Insurance disparity may affect the choice of treatment for patients with ESRD, as shown in the United States [11], South America [18] and Australia [19]. However, another study has shown that the economic status was not independently associated with outcomes in a large multicenter Brazilian cohort [12]. The primary outcome of the present study was to assess the association between insurance type and survival in PD patients, and results showed that PD patients with NCMS had a lower survival compared with PD patients with UEMI. Many factors might be responsible for this observation, such as education, nutrition, distance between home and hospital. Results of the present study are supported by a study from Columbia that showed that PD and hemodialysis patients with poor medical insurance coverage had lower survival [20]. Most importantly, the present study showed that the mortality due to financial reasons was higher in patients with NCMS compared with UEMI, underlining the importance of economics and insurance coverage in ESRD survival.

Previous studies in China showed that a high school education or higher was less frequent in rural areas and that the prevalence of CKD was higher compared with urban patients [21]. The present study showed that in accordance with their economic status, rural patient had a low education level. In addition, rural patients were younger, which might be related to a lower awareness of CKD, which may delay treatments in these patients and ultimately allow CKD to progress to ESRD.

Previous studies showed that Chinese rural patients have a lower proportion of hypertension and diabetes control [22,23]. In the present study, fewer rural patients suffered from diabetic nephropathy, and their diastolic BP control was inferior compared with urban patients, which is similar to previous epidemiological data from China [22,23].

Low hemoglobin levels, hyperphosphoremia, malnutrition and lower residual renal function are predictors of mortality in PD patients, and these predictors are independent from gender, race and dialysis buffer [6,24-26]. In our center, we observed that patient with NCMS had lower hemoglobin levels compared with patients with UEMI, which might be a consequence of malnutrition due to a lower economic status. In addition, these patients are less likely to be able to afford intravenous iron and erythropoiesis-stimulating agent therapy. Foods with high protein content are also an important source of dietary phosphorus [27]. However, even if patients with NCMS are able to consume high-protein foods, they usually are unable to afford phosphorus binders such as sevelamer carbonate and lanthanum carbonate. As a result, hyperphosphatemia was more common in rural patients even if albumin levels and nPCR were not significantly different between the two groups. Finally, we observed no difference on residual renal function between the two groups, and we presume that it may be related to the longer dialysis duration for patients with UEMI.

Studies have shown that mortality was higher among PD patients who lived farther from their attending nephrologist compared with patients who lived closer, which may be the result of less frequent contact with a nephrologist for patients in remote areas [28-30]. However, another study has shown that although the risk of death was significantly higher in remote dwellers, distance from the dialysis center and residing in rural areas did not affect the risk of technique failure or death [31]. In China, patients with NCMS mainly live in rural areas, which are often underdeveloped areas far from any hospital compared with patients with UEMI. They live farther from their treating center and have less contact with their physician. Dialysis inadequacy is an important predictor for mortality in PD patient, especially for anuric patients [32]. Studies have shown that a Kt/V <1.7 was associated with an increased risk of mortality and hospitalization, and that this observation persisted after multivariate adjustment [33]. The present study suggests that although the dialysis volume was lower in rural patients compared with urban patients, the Kt/V and CrCL were not different between the two groups, indicating that the different clinical outcomes were not associated with solute clearance.

Previous studies have shown that the main causes of switching from PD to HD were infections, fluid overload, abdominal surgery, malnutrition, decreased mental capacity and abdominal wall defect [34]. Despite a probable higher rate of malnutrition among rural patients compared urban patients, we did not observe any difference in the rate of switching to HD. This may be because the switching rate is affected by many factors, and because we did not assess all of them in the present study. Further studies are needed to assess this specific issue. Nevertheless, a likely explanation might be the lack of access to a HD facility in remote rural areas. As for the similarity in transplantation rate, the limited access to compatible donor kidneys might be a limiting factor that is more important than financial reasons.

Peritonitis is the most frequent cause of peritoneal dialysis failure, and significantly affects patient mortality [35]. However, it is a modifiable factor and the patients may be switched to hemodialysis [36]. In addition, a previous study has shown that living more than 100 km from a dialysis unit increased the risk of *S. aureus* peritonitis [13]. In the present study, data from patients with technique failure showed that peritonitis was the major cause of switching to hemodialysis, and that there was no difference between the two groups. Therefore, the higher rate of treatment cessation in patients with NCMS may be related to the lower survival. In addition, results showed that DN and hypoalbuminemia were independent predictors for technique failure.

This study has several limitations. First, data were from a single center and the sample was small, preventing extrapolation of the results to all Chinese patients with ESRD and to other countries. We did not evaluate the comorbidities and previous treatments at enrollment. We did not evaluate the distances between the patient, his family and the hospital. Finally, the cause of death for many patients had to be presumed by the nephrologist, especially for the patients who died at home.

## Conclusions

In summary, biomedical parameters were inferior in patients with NCMS compared with patients with UEMI. Although the technique failure rate was similar between the two groups, survival was lower in patients with NCMS. Therefore, in light of our findings, we suggest that the medical coverage rate for patients with ESRD needs to be improved. Health policy makers should consider building satellite PD centers and developing PD networks to standardize the training programs. For the nephrologists, there is a need to reinforce targeted intervention for rural patients and to implement suitable treatments based on their economic status and insurance type.

## Abbreviations

BP: Blood pressure
BMI: Body mass index
CKD: Chronic kidney disease
CrCL: Creatinine clearance
ESRD: End-stage renal disease
GFR: Glomerular filtration rate
HD: Hemodialysis
PD: Peritoneal dialysis
NCMS: New Cooperative Medical Scheme
UEMI: Urban Employees’ Medical Insurance
